# Influence of Antiplatelet Agents on the Lipid Composition of Platelet Plasma Membrane: A Lipidomics Approach with Ticagrelor and Its Active Metabolite

**DOI:** 10.3390/ijms22031432

**Published:** 2021-01-31

**Authors:** Jennifer Lagoutte-Renosi, Florentin Allemand, Christophe Ramseyer, Vahideh Rabani, Siamak Davani

**Affiliations:** 1 EA 3920, Université Bourgogne Franche-Comté, F-25000 Besançon, France; jennifer.lagoutte_renosi@univ-fcomte.fr (J.L.-R.); v.rabani@gmail.com (V.R.); 2 Laboratoire de Pharmacologie Clinique et Toxicologie-CHU de Besançon, F-25000 Besançon, France; 3 Laboratoire Chrono Environnement UMR CNRS 6249, Université de Bourgogne Franche-Comté, 16 Route de Gray, CEDEX, 25030 Besançon, France; florentin.allemand@edu.univ-fcomte.fr (F.A.); christophe.ramseyer@univ-fcomte.fr (C.R.)

**Keywords:** lipidomics, platelets, plasma membrane, lipids, receptor, ticagrelor

## Abstract

Lipids contained in the plasma membrane of platelets play an important role in platelet function. Modifications in the lipid composition can fluidify or rigidify the environment around embedded receptors, in order to facilitate the access of the receptor by the drug. However, data concerning the lipid composition of platelet plasma membrane need to be updated. In addition, data on the impact of drugs on plasma membrane composition, in particular antiplatelet agents, remain sparse. After isolation of platelet plasma membrane, we assessed, using lipidomics, the effect of ticagrelor, a P2Y12 antagonist, and its active metabolite on the lipid composition of these plasma membranes. We describe the exact lipid composition of plasma membrane, including all sub-species. Ticagrelor and its active metabolite significantly increased cholesterol and phosphatidylcholine ether with short saturated acyl chains 16:0/16:0, and decreased phosphatidylcholine, suggesting overall rigidification of the membrane. Furthermore, ticagrelor and its active metabolite decreased some arachidonylated plasmalogens, suggesting a decrease in availability of arachidonic acid from the membrane phospholipids for synthesis of biologically active mediators. To conclude, ticagrelor and its active metabolite seem to influence the lipid environment of receptors embedded in the lipid bilayer and modify the behavior of the plasma membrane.

## 1. Introduction

Blood platelets play a pivotal role as regulators of haemostasis and thrombosis [1]. The platelet plasma membrane, composed of a lipid bilayer with embedded proteins, is the support for adhesion, activation and aggregation of platelets through receptors [2]. G-protein coupled seven-transmembrane P2Y12 receptors belonging to the transmembrane proteins are present in the platelet plasma membrane. These receptors are implicated in adenosine diphosphate (ADP)-induced activation of platelets and, therefore, constitute a prime target to inhibit platelet aggregation [3]. Platelet aggregation contributes to the onset of acute coronary syndrome, and current guidelines recommend dual antiplatelet therapy, including a P2Y12 antagonist and aspirin, for at least 12 months after the cardiovascular event [4].

In this context, P2Y12 antagonists such as clopidogrel, ticagrelor and prasugrel were developed to prevent cardiovascular events, especially after acute coronary syndrome. Among the available P2Y12 antagonists, ticagrelor has been widely used because of its superior efficacy, with less inter-individual variation and faster onset of action than clopidogrel for the control of platelet aggregation [4,5]. This is probably related to its additional mechanism of action by the inhibition of Equilibrative Nucleoside Transporter 1 (ENT1) increasing circulating and tissues levels of adenosine [6,7]. 

Unlike other P2Y12 antagonists, ticagrelor is not a pro-drug and does not require hepatic biotransformation to be active. However, ticagrelor is also metabolized into an active metabolite AR-C124910XX, which possesses similar potency to the parent ticagrelor [6]. We recently showed that platelet stimulation by ADP, or platelet inhibition by ticagrelor, changes the organization of the platelet plasma membrane, with reorganization of cholesterol and P2Y12 receptors to lipid rafts. This reorganization suggests that membrane lipids, in particular cholesterol, play an important role in the anti-aggregant activity of ticagrelor [8]. Despite the existence of abundant literature on ticagrelor/P2Y12 receptor interactions, the direct effects of ticagrelor and its active metabolite AR-C124910XX on the lipid composition of platelet plasma membrane remain to be determined. 

It has been shown that changes in lipid composition induce structural and functional changes in plasma membranes [9], so we aimed to describe as accurately as possible the lipid composition of the platelet plasma membrane, including details of the different head groups, fatty acyl chain length and saturation, both in resting platelets and after ticagrelor treatment. Lipidomics has previously been used to investigate plasma and subcellular membranes of whole platelets [10,11] but no lipidomic data are available for platelet plasma membranes only. 

Using advances in lipidomics, we first provide up-to-date data regarding the lipid profile of the platelet plasma membrane and, secondly, we investigated the lipid changes occurring in the platelet plasma membrane in the presence of ADP, ticagrelor or its active metabolite. 

## 2. Results

### 2.1. VASP Analysis

Inhibition of platelets in vitro was assessed with different concentrations using the vasodilatator-stimulated phosphoprotein (VASP) index, which determines the platelet reactivity index. Efficient platelet inhibition is obtained when the platelet reactivity index (PRI) is below 50% [12]. We obtained a PRI of 29.5% for 1.5-µM ticagrelor, and 27.4% for 0.5-µM active metabolite. Therefore, concentrations of 1.5 µM of ticagrelor and 0.5 µM of active ticagrelor metabolite were assumed to achieve excellent platelet inhibition and were used for all subsequent lipidomics experiments.

### 2.2. Immunoblot Analysis

**Figure 1:**
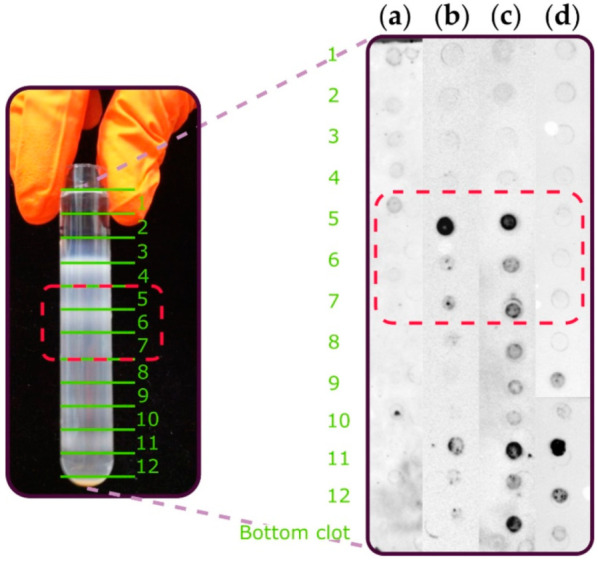
Specific protein markers assessed by immunoblot. (**a**) The neutrophil cytosolic factor 1 (NCF1) marker of cytosol was present in the first four fractions. (**b**) The Flotillin-1 marker of cholesterol-rich microdomains was mainly detected in fractions 5–7 of sucrose. (**c**) The glycoprotein Ib (GPIb) marker of platelet plasma membrane was present in fractions 5–12 and (**d**) the cytochrome c oxidase IV (COX IV) marker of mitochondria was mainly present in fractions 9–12.

In order to ensure the position of the platelet plasma membrane in sucrose fractions, the presence of specific protein markers was evaluated by immunoblot assay in the control group. Our results showed that neutrophil cytosolic factor 1 (NCF1), a specific marker of cytosol, was weakly present in the first five fractions and not expressed in fractions 6–12 (Figure 1a). Flotillin-1, a marker of cholesterol-rich microdomains, was mainly expressed in fractions 5–7 (Figure 1b). Glycoprotein Ib (GP1b) was found in fractions 5–12 due to its abundance as a principal protein of all intracellular platelet membranes, including plasma and organelle membranes (Figure 1c). Finally, cytochrome c oxidase IV (COX IV), a marker of mitochondria, was positive mainly in fractions 9–12. Taken together, we considered that fractions 5–7 contained plasma platelet membrane and fractions 8–12 contained membranes of mitochondria, lysosome and granules (Figure 1d). Consequently, fractions 5–7 were analyzed using lipidomics.

### 2.3. Lipid Composition of Plasma Membrane in Resting Platelets

Mass spectrometry-based lipid analysis was performed in the control group. Our results showed that the plasma membrane of resting platelets was mainly composed of phosphatidylcholines (PC): 35.2 ± 0.8%; cholesterol (Chol): 28.35 ± 0.7%; phosphatidylethanolamines ether (PE O-): 10.40 ± 0.2%; sphingomyelins (SM): 7.26 ± 0.3%; phosphatidylserines (PS): 6.06 ± 0.1%; phosphatidylethanolamines (PE): 6.41 ± 0.1%; phosphatidylinositols (PI): 2.41 ± 0.03% and phosphatidylcholines ether- (PC O-): 1.84 ± 0.04%. These results were compared to others in Table 1. Other lipid classes are also present in much smaller amounts (see Supplementary Materials Table S1).

**Figure 2:**
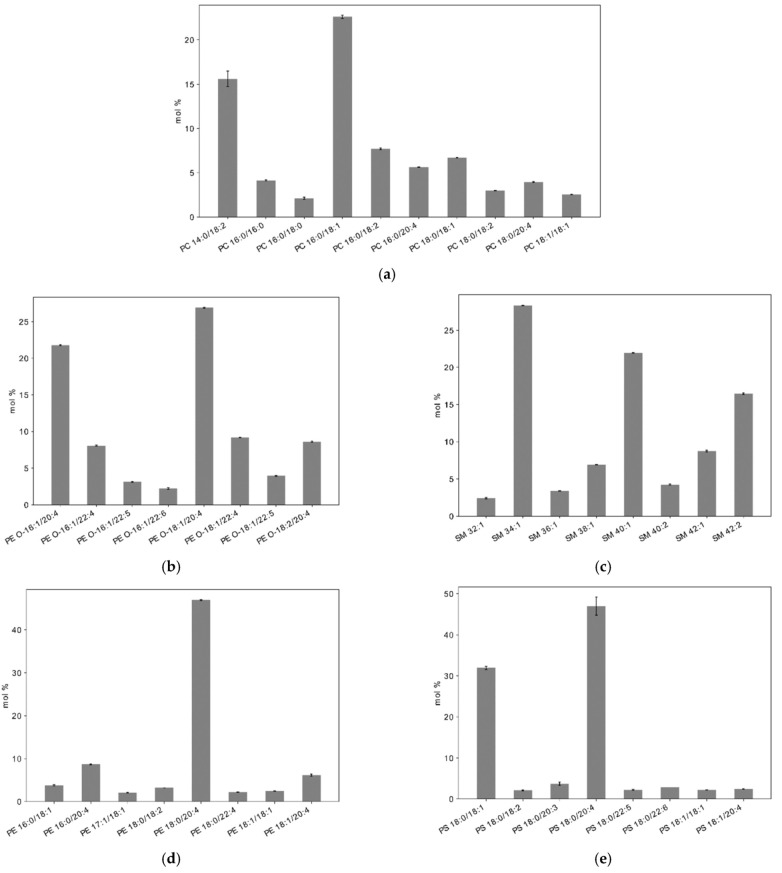

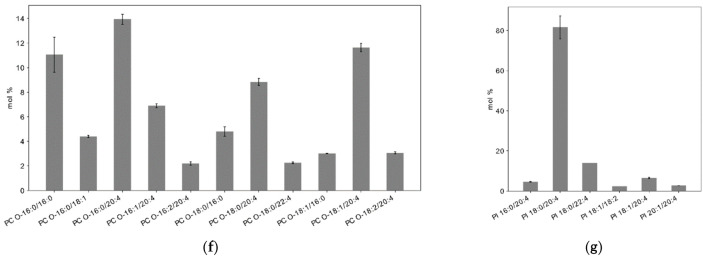
Lipid composition of the plasma membrane of resting platelets with (**a**) Phosphatidylcholines (PC), (**b**) Phosphatidylethanolamines ether (PE O-), (**c**) Sphingomyelins (SM), (**d**) Phosphatidylethanolamines (PE), (**e**) Phosphatidylserines (PS), (**f**) Phosphatidylcholines ether (PC O-) and (**g**) Phosphatidylinositols (PI). Results are expressed in mol% inside each sub-class of lipids. Lipids below 2% of its sub-classes were not represented. Complete values are available in supplementary data (Table S1).

Since the size of headgroups, length and saturation of acyl chains of lipids are responsible for the physicochemical properties of the plasma membrane, we assessed in resting platelets the exact composition of lipids in the platelet plasma membrane, with size, length and saturation of their acyl chains in each subclass. In summary, in the plasma membrane, our results showed that short fatty acyl chains, with 14, 16 or 18 carbons, are predominant in all subclasses (Figure 2). Arachidonic acid (20:4) is also well-represented, especially in PC O- and PE O-, which contain plasmalogens, that play an important role both for the structure of plasma membrane and for cell signalling. Concerning the fatty acid composition of the platelet plasma membrane, the main saturated fatty acids were palmitic acid 16:0 (17.1 ± 0.2%) and stearic acid 18:0 (14.9 ± 0.1%), whereas the main unsaturated fatty acids were oleic acid 18:1 (19.3 ± 0.02%), arachidonic acid 20:4 (18.0 ± 0.2%), and linoleic acid 18:2 (10.6 ± 0.2%) (Figure 2 and Table S1). Eicosapentaenoic acid 20:5 (EPA) and Docosahexaenoic acid 22:6 (DHA) are minor among the acyl chain of phospholipids, with 0.49 ± 0.01 % and 0.79 ± 0.04%, respectively, (Figure 2 and Table S1). 

**Figure 3:**
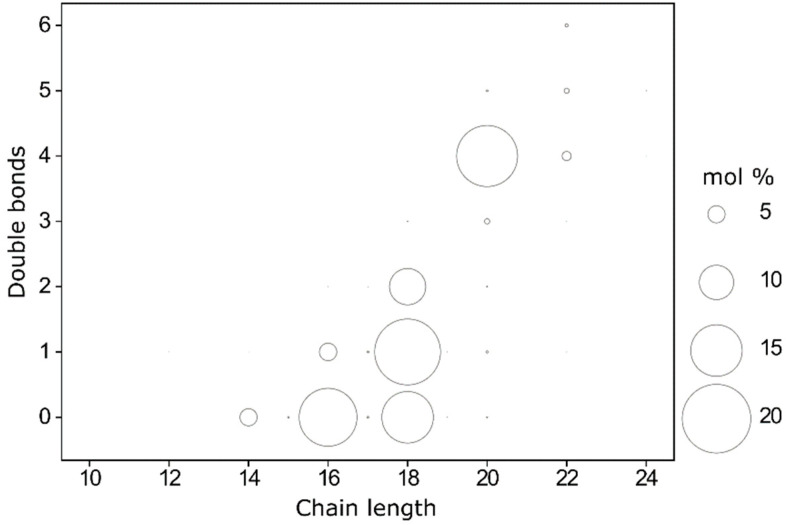
Fatty acid species in the plasma membrane of resting platelets represented by chain length and number of double bonds. The area of each circle is proportional to the abundance of each fatty acid in mol%.

Among the phospholipids, 61.56% of their fatty acids were unsaturated, with an unsaturated/saturated ratio of fatty acids of 1.60. Polyunsaturated fatty acids (PUFA) represented 35.9 ± 0.1% of these fatty acids (Figure 3).

### 2.4. Impact of ADP, Ticagrelor and Its Active Metabolite on Lipid Classes of Plasma Membrane 

**Figure 4:**
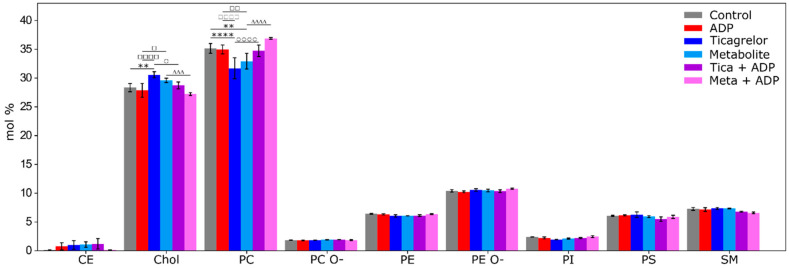
Lipid composition of the platelet plasma membrane according to the experimental groups. The results are expressed as mol% (mean ± SD) of lipid sub-classes. Cholesteryl esters (CE), cholesterol (Chol), phosphatidylcholines (PC), phosphatidylcholines ether (PC O-), phosphatidylethanolamines (PE), phosphatidylethanolamines ether (PE O-), phosphatidylinositols (PI), phosphatidylserines (PS), sphingomyelins (SM). Lipids below 1% of their sub-class are not represented. *p*-values were determined from two-way ANOVA (* = *p* < 0.05, ** = *p* < 0.01, *** = *p* < 0.005, **** = *p* < 0.001 compared to the control group; □ = *p* < 0.05, □□ = *p* < 0.01, □□□□ = *p* < 0.001 compared to the adenosine diphosphate (ADP) group, ○ = *p* < 0.05, ○○○○ = *p* < 0.001 compared to the Ticagrelor group; ∆∆∆ = *p* < 0.005, ∆∆∆∆ = *p* < 0.001 compared to the Metabolite group).

For comparison of the effect of ADP, ticagrelor and its active metabolite on the platelet plasma membrane, we chose first to focus on the main sub-classes of lipids, represented by a percentage above 1% of the total amount of lipids in the plasma membrane, and second, to investigate changes in each sub-class, especially in different fatty acyl chains of lipids (Figure 4). Principal component analysis (PCA) with data for the lipid species showed that replicates are close to each other. Accordingly, all replicates were included in the analysis.

The distribution of the main lipid classes in the platelet plasma membrane showed significant differences between experimental groups for two major classes, namely cholesterol and PC (Figure 4). Cholesterol was significantly more present in the Ticagrelor (30.6 ± 0.5%) compared to control groups (28.4 ± 0.7%, *p* < 0.001) and in Metabolite (29.6 ± 0.4%) compared to ADP groups (27.9 ± 1.2%, *p* < 0.05). PC were significantly less present in the Ticagrelor (31.7 ± 1.8%) compared to the control groups (35.2 ± 0.8%, *p* < 0.001) and in Metabolite (32.9 ± 1.4%) compared to ADP groups (35.0 ± 0.8%, *p* < 0.01). For cholesterol and PC, when platelets were pre-treated by ticagrelor or its active metabolite, stimulation by ADP induced a return to the initial lipid composition found in the control group or ADP treated platelets. A small amount of cholesteryl esters was found in all groups. This can be explained by the interaction of lipoproteins with the surface of the platelets, especially in the open canalicular system of the platelet [24]. No significant difference was found for PC O-, PE O-, PE, PI, PS, or SM between experimental groups (Figure 4).

**Figure 5:**
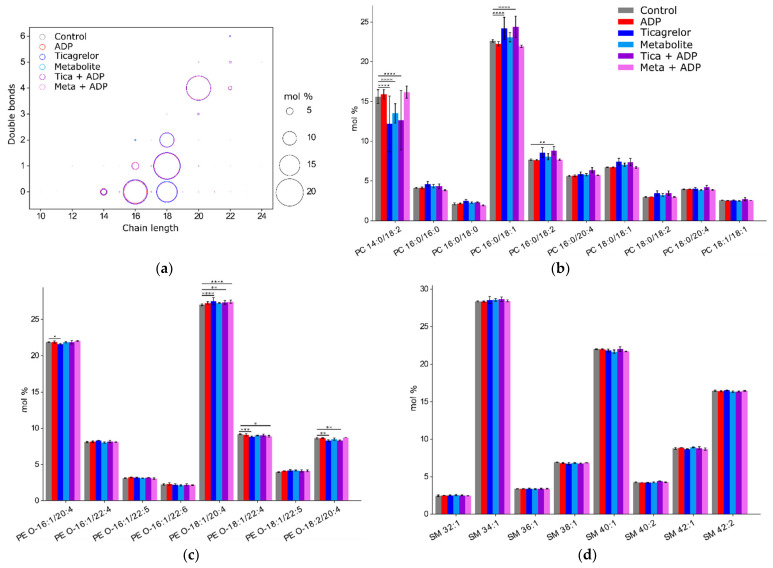

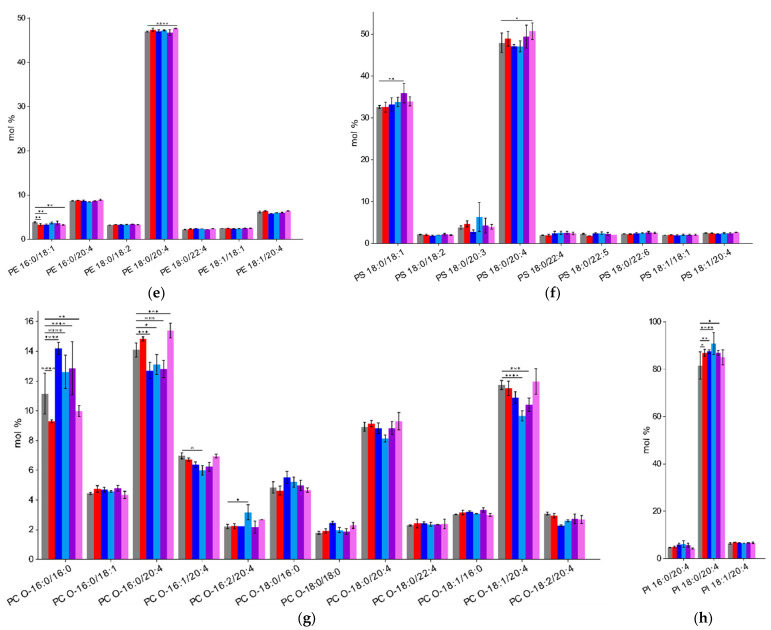
Composition of the platelet membrane. (**a**) Fatty acids species in the platelet plasma membrane represented by chain length and number of double bonds. The area of each circle is proportional to the abundance of each fatty acid in mol%. Subspecies which represent more than 2% of (**b**) phosphatidylcholines (PC), (**c**) phosphatidylethanolamines ether (PE O-), (**d**) sphingomyelins (SM), (**e**) phosphatidylethanolamines (PE), (**f**) phosphatidylserines (PS), (**g**) phosphatidylcholines ether (PC O-) and (**h**) phosphatidylinositols (PI). *p*-value was determined by a two-way ANOVA test (* = *p* < 0.05, ** = *p* < 0.01, *** = *p* < 0.005, **** = *p* < 0.001 versus the control group).

However, lipid classes were composed of many subspecies with a variety of fatty acid chain lengths, so we investigated these subspecies to assess whether some subspecies were different between experimental groups, and if the significant difference in PC was due to a particular subspecies (Figure 5a). In the PC class, PC 14:0/18:2 decreased significantly in the Ticagrelor (12.2 ± 3.5 %) and Metabolite groups (13.5 ± 1.2%) compared to the control group (15.6 ± 0.9%, *p* < 0.001 and *p* < 0.001, respectively), whereas PC 16:0/18:1 increased significantly in the Ticagrelor (24.2 ± 1.4%) compared to the control group (22.6 ± 0.2%, *p* < 0.001) (Figure 5b). 

In the PE O- class, PE O- 18:1/20:4 increased significantly in the Ticagrelor (27.5 ± 0.5%) versus the control group (27 ± 0.1%, *p* < 0.001) (Figure 5c). In contrast, PE O- 16:1/20:4 and PE O- 18:2/20:4 were significantly decreased in the Ticagrelor group (21.6 ± 0.09% and 8.3 ± 0.1,) versus the control group (21.9 ± 0.04%, *p* < 0.05 and 8.6 ± 0.1%, *p* < 0.01) (Figure 5c). 

For the SM class, no significant differences were found between groups (Figure 5d). 

In the PE class, PE 16:0/18:1 was higher in the control (3.8 ± 0.2%) than in the ADP (3.3 ± 0.3%, *p* < 0.01), Ticagrelor (3.3 ± 0.2%, *p* < 0.01) and Meta + ADP (3.3 ± 0.1%, *p* < 0.01) groups. PE 18:0/20:4 was higher in the Meta + ADP (47.7 ± 0.1%) than in the control group (47.0 ± 0.1%, *p* < 0.001) (Figure 5e).

In the PS class, PS 18:0/18:1 was higher in the Tica + ADP (35.9 ± 2.3%) than in the control group (32.6 ± 0.4%, *p* < 0.01). PS 18:0/20:4 was higher in the Meta + ADP (50.7 ± 2.0%) than in the control group (47.9 ± 2.4%, *p* < 0.05) (Figure 5f). 

PC O- with short acyl chains such as 16:0/16:0 increased significantly in the Ticagrelor (14.2 ± 0.4%) and metabolite groups (12.6 ± 1.1%) versus the control group (11.2 ± 1.4%, *p* < 0.001 and *p* < 0.001, respectively), whereas PC O- with arachidonic acid, 16:0/20:4 decreased significantly in the Ticagrelor (12.7 ± 0.5%) and metabolite groups (13.1 ± 0.7%) versus the control group (14.1 ± 0.5%, *p* < 0.005 and *p* < 0.05, respectively) (Figure 5g). In the Metabolite group, several PC O- species containing arachidonic acid 16:1/20:4, 18:1/20:4, 16:2/20:4 were significantly decreased (6.0 ± 0.3%; 9.7 ± 0.4% and 3.2 ± 0.5%) compared to the control group (7.0 ± 0.2%, *p* < 0.05; 11.7 ± 0.3%, *p* < 0.001 and 2.2 ± 0.1%, *p* < 0.05, respectively) (Figure 5g). In the PI class, PI 18:0/20:4 was significantly increased in the Ticagrelor (87.5 ± 0.7%) and metabolite groups (90.8 ± 4.6%) compared to the control group (81.6 ± 5.7%, *p* < 0.01 and *p* < 0.001, respectively) (Figure 5h).

Plasmalogens, such as PUFA containing arachidonic acid in their acyl chains were significantly decreased for both ticagrelor and its metabolite (PC O- 16:0/20:4, PC O- 18:1/20:4), or for ticagrelor only (PE O- 16:1/20:4, PE O- 18:2/20:4).

**Figure 6:**
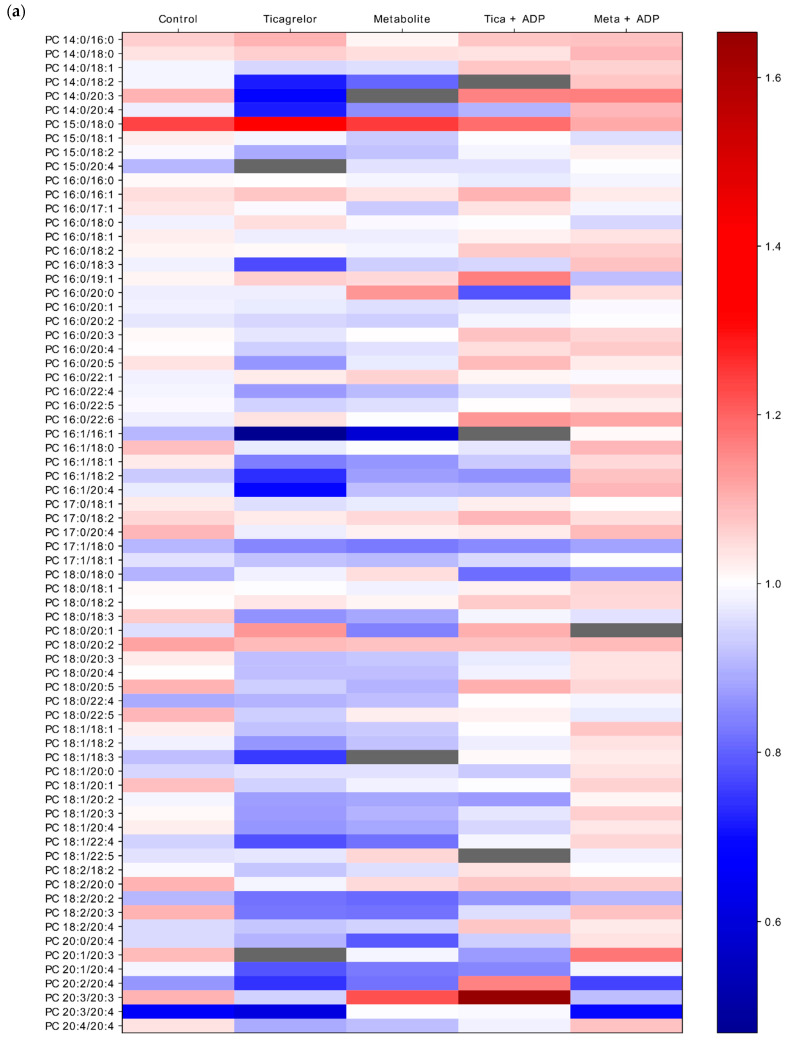

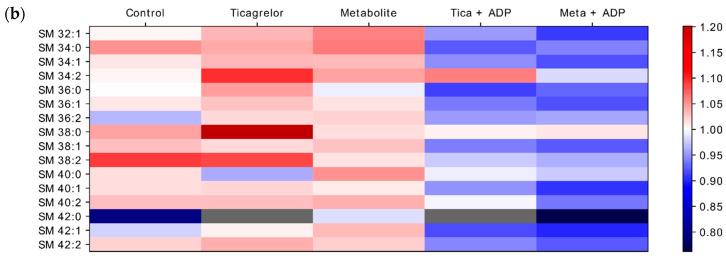
Heatmap representing fold change in (**a**) Phosphatidylcholines (PC) or (**b**) Sphingomyelins (SM) subspecies compared to the ADP group, according the experimental groups. When platelets were pre-treated by ticagrelor or its active metabolite, stimulation by ADP did not lead to an SM or PC composition similar to ADP-isolated stimulation but led to an increase in PC and a decrease in SM fold change compared to the ADP group.

The amount of PC and SM subspecies are shown in the heatmap (Figure 6a,b). Stimulation by ADP did not lead to a return to a PC composition similar to ADP-isolated stimulation but resulted in an overall increase in all PC species (Figure 6a). The reverse phenomenon was observed with SM subspecies. Indeed, when platelets were pre-treated by ticagrelor or its active metabolite, stimulation by ADP did not lead to a composition similar to ADP-isolated stimulation, but, in contrast, we observed an overall decrease in all SM (Figure 6b). 

**Figure 7:**
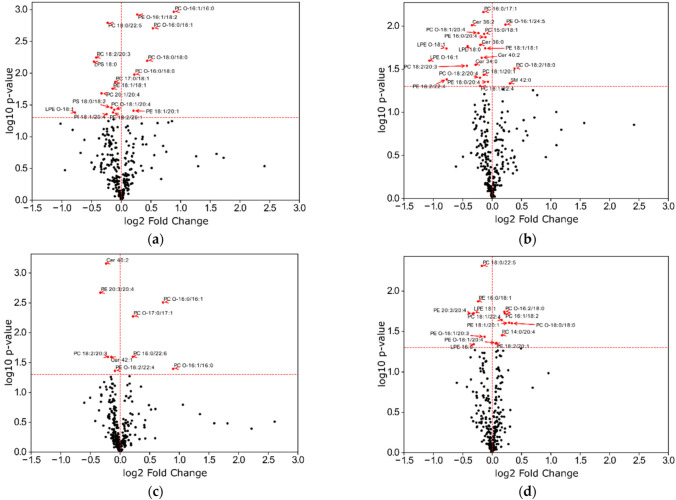

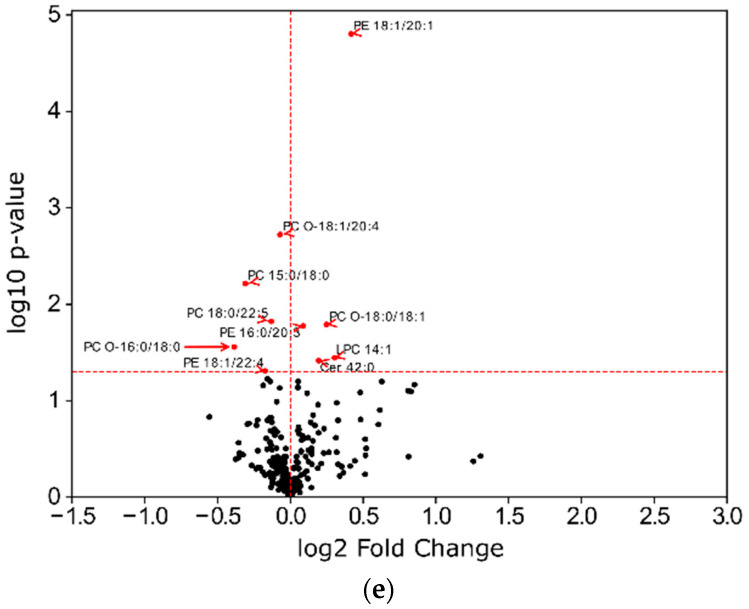
Volcano plot of lipid subspecies of the control group versus groups treated by (**a**) Ticagrelor, (**b**) Metabolite, (**c**) Tica + ADP, (**d**) Meta + ADP and (**e**) ADP. *p*-values were calculated by one way ANOVA. The dashed red horizontal line represents *p*-value > 0.05.

To investigate whether treatment induced more significant variations in the number of specific lipids, we decided to build volcano plots and to compare the control group to other experimental groups (Figure 7). 

We noted that a greater number of lipid species were significantly different in the Ticagrelor (Figure 7a) and Metabolite (Figure 7b) groups compared to the Tica + ADP (Figure 7c) and Meta + ADP (Figure 7d) groups. Thus, treatment by drugs led to a greater number of lipid species having significantly different amounts, in the plasma membrane than in the ADP (Figure 7e), Tica + ADP, and Meta + ADP groups. 

## 3. Discussion

To the best of our knowledge, this is the first study to describe the lipid composition of plasma membrane of resting platelets using advances in lipidomics. Moreover, our study is the first to investigate the lipid changes in the plasma platelet membrane after stimulation of platelets by ADP, or inhibition by antiplatelet agents such as ticagrelor and its active metabolite. Our major findings were, (i) the precise determination of the lipid composition of plasma membrane of resting platelets, (ii) a significant increase in cholesterol and PC O- with short acyl chains 16:0/16:0 in platelet plasma membrane treated with ticagrelor and its metabolite, (iii) a significant decrease in PC and some PC O- or PE O- enriched in arachidonic acid (PE O- 16:1/20:4, PE O- 18:2/20:4, PC O- 16:0/20:4, PC O- 18:1/20:4) with ticagrelor, (iv) pre-treatment by ticagrelor and its active metabolite induces a long-lasting effect over time on lipid composition, especially for PC and SM even if ADP is present. 

Describing as accurately as possible the lipid composition of the platelet plasma membrane, including details of the different properties of lipids such as head groups, fatty acyl chain length and saturation, is key to understanding the structural and functional changes in the platelet plasma membrane [9]. The structure of human eukaryotic plasma membranes has been extensively investigated. Indeed, human erythrocytes were used as a simple model because of their lack of nucleus and internal organelles [25]. Moreover, erythrocytes can easily be collected from individuals for many experiments. Although the composition of red blood cells is relatively well described, other type of cells such as platelets remain poorly detailed in the literature. Few data are available concerning the composition of the platelet plasma membrane. In addition, previous studies are quite old and used less sensitive and specific techniques, explaining the variations between studies (Table 1). Furthermore, previous determinations of lipid composition in plasma membrane were limited to the few major lipid types defined by their polar headgroups. Advances in lipidomics have revealed a vast diversity of lipid species in cell membranes, including hundreds of lipid subspecies with distinct headgroups, acyl chains, and backbone linkages [26]. With our study, we provide updated data using new lipidomics techniques and summarize all pre-existing data in the literature in Table 1. 

Unlike previous studies, we provide up-to-date data not for plasma and the subcellular membrane of the whole platelet [17,18,19,20,21], but rather only for the platelet plasma membrane, including the cholesterol amounts. Indeed, several studies explored only major phospholipids grouped by their polar headgroup and did not include cholesterol analysis in the plasma membrane [13,14,15,16]. Tsvetkoya et al. [15] described the lipid composition using rod like thin layer chromatography in resting platelet plasma membrane including cholesterol. They reported 7.5% of cholesterol, which is surprising because typically most cell membranes have a cholesterol level between 10 and 30% of all lipids [27]. In our study, resting platelet plasma membrane was composed of 28.35% cholesterol, which is coherent for a cell membrane, but one of the highest values described in the literature for platelets. This difference could be explained by the use of mass tandem spectrometry, which is more sensitive and more specific than thin layer chromatography and gas–liquid chromatography. 

Our study also distinguished PC, PC O-, PE and PE O-. Previous studies using thin layer chromatography and gas–liquid chromatography probably did not distinguish between PC/PC O-, and PE/PE O-. It is for this reason that our study found a lower level of PC and PE than other studies, but if we add the PC O-, or PE O- within the same subclasses of PC and related species or PE and related species, our results are in accordance with studies using thin layer chromatography. However, sphingomyelins were less abundant than expected in the platelet plasma membrane. Several studies investigated the lipidome of whole platelets, without quantification of cholesterol [17,18,19], except Owen et al. [20], Dougherty et al. [21] and Leidl et al. [23]. These studies showed that the whole lipidome of platelet contained a similar proportion of cholesterol, i.e., approximately 30%, less PC and related PC and higher sphingomyelin levels than our results on the plasma membrane. Concerning the fatty acid composition of phospholipids, Watanabe et al. [22] showed that major saturated fatty acids on platelet plasma membrane were 17% palmitic acid, 21.3% stearic acid, and major unsaturated fatty acids were 22% arachidonic acid, 17.1% oleic acid, 6% linoleic acid, 2.5% DHA, and 2% EPA. These values are in accordance with our results, although we found that unsaturated fatty acids were more abundant, with oleic and linoleic acid being more present, while arachidonic acid was less present. PUFAs are related to important biological functions in health and disease, especially in cardiovascular diseases [28]. In the plasma membrane, phospholipids containing PUFAs decrease membrane bending rigidity [29] and render the membrane more flexible [9], by reducing the energy required for deformation and fission [30]. 

Potential changes in the lipid composition of the platelet plasma membrane cause structural and functional changes in the platelet plasma membrane. Indeed, the lipid composition of plasma membrane changes these physicochemical properties. Lipids have intrinsic shapes depending on the size of their headgroups, length and saturation of their acyl chains [31]. Therefore, modification of the lipid composition affects the shape and the spontaneous curvature of the plasma membrane [29,31] and its flexibility [9]. Lipids with long and saturated fatty acids render membranes thicker and less fluid owing to the tight packing of their hydrophobic tails and stronger lipid–lipid interactions. Conversely, lipids with unsaturated fatty acids prevent tight packing because of acyl-chain kinks [29]. Thus, plasma membranes are defined by regions with more or less liquid packing, named liquid ordered domains and liquid disordered domains, respectively [29]. Liquid ordered domains contain microdomains enriched in cholesterol, relatively saturated lipids and sphingolipids, which act as a functional platform to recruit other lipids and proteins and regulate cellular functions [32,33]. These microdomains promote protein–protein and lipid–protein interactions [34]. Lipid–protein interactions have been garnering increased interest; in particular, the modulation of the biophysical properties of the plasma membrane by lipids and key functional roles of specific lipids [35]. Thus, lipid–protein interactions are influenced by the modification of the lipid composition, which implies a modification of the shape, the physicochemical behavior and the functional properties of the plasma membrane, therefore modifying the environment of the target receptor. 

Previously, lipid changes in platelet plasma membrane were described with platelet activators. Thrombin, a human platelet activator, caused significant alteration in lipids of platelet plasma membrane with a decrease in PI of up to 45%. In platelets activated by thrombin, the proportion of PI and PC with arachidonic acid decreased, which suggests that PI and PC enriched in arachidonic acid were the source of the eicosanoid synthesis [14,36]. In our study, the impact of ADP-induced activation on lipid changes in the platelet plasma membrane was investigated and compared to ticagrelor. In this study, we used only a single concentration of ADP (20 µM) to achieve effective and total platelet aggregation. This concentration was chosen in accordance with the manufacturer’s instructions in order to avoid partial or delayed activation. However, this choice could be considered as a limitation of the experimental study. Surprisingly, no major changes in membrane lipid composition were observed between control and ADP stimulated platelets. However, when platelets where pre-treated by ticagrelor or its active metabolite before stimulation by ADP, a decrease in SM and an increase in PC can be observed compared to the ADP group. Thus, interestingly, pre-treatment by inhibitors before ADP stimulation did not lead to a return to the lipid profile similar to the ADP group. Ticagrelor and its active metabolite seem to have a long-term effect on the lipid composition even if an agonist such as ADP is once again present to stimulate platelets. These results suggest that the effect of ticagrelor and its active metabolite on the lipid composition of platelet plasma membrane is long-lasting.

Changes in the lipid profile of the platelet plasma membrane have also been described in several diseases, but drug-induced modifications have been rarely investigated to the best of our knowledge. Watanabe et al. showed that PS and PI decreased significantly in platelet plasma of patients with alcoholic liver disease [22]. Palmitic acid and EPA were also significantly decreased in the platelet plasma membrane of these patients. Prisco et al. reported that platelet lipid composition was altered in neoplastic patients with pulmonary cancer, in particular with a decrease in PC and PE enriched in linoleic acid and n-3 PUFA esterified, such as 20:5; 22:5; 22:6 unsaturated acyl chains [37]. Interestingly, Ren et al. also showed a decrease in PE enriched in linoleic acid and 22:6 n-3 (DHA) as well as a greater proportion of arachidonic acid in whole platelets of patient with homozygous sickle cell disease versus healthy patients [38]. Recently, García-Rubio et al. showed that platelet plasma membrane from patients with arterial hypertension also presented lipid alterations, including phosphatidylcholine and cholesterol depletion [39]. In the same manner, we observed here a depletion of phosphatidylcholine in plasma membrane after treatment of platelets by ticagrelor and its active metabolite. Thus, regulation of the lipid composition of platelet plasma membrane seems complex and could be impacted by both pathologies and drugs. Exploration of these modifications by drugs are still limited but are essential to better understand the potential reorganization of the plasma membrane structure constituting the environment of the target receptor of the drugs. Indeed, molecular dynamics studies confirmed the role of membranes in modulating proteins’ conformational state [35,40,41]. Modifications of the lipid composition lead to fluidifying, rigidifying, stabilizing environment around embedded receptors, in order to facilitate the access to the receptor by the drug. Interestingly, Mahmood et al. showed that lipid composition affects conformation of β2 adrenergic receptor which is a G-protein coupled receptor, in the same way as P2Y12 [41]. 

Few studies have demonstrated the impact of drugs on the plasma membrane, either directly by modification of the lipid composition [8], or indirectly, by modification of the fluidity of the membrane [42]. Conversely, the lipid composition of the plasma membrane could impact the behavior of drugs, especially the incorporation of the drug into the lipid membrane [43,44]. Thus, aspirin, an antiplatelet agent, induced a reduction in membrane lipid fluidity, independently of its acetylating effect on platelet cyclo-oxygenase [42,45]. This result is in line with our study showing that ticagrelor increases cholesterol level, inducing an overall rigidification of the plasma membrane, with a decrease in membrane fluidity close to the bilayer surface and an increase in fluidity near the bilayer center [27]. 

Cholesterol is a key molecule in controlling membrane fluidity. It exhibits a stronger stiffening effect in the presence of saturated fatty acids than in the presence of unsaturated fatty acids [46]. Here, ticagrelor increases the cholesterol level and ether phosphatidylcholines enriched in short, saturated acyl chains such as PC O- 16:0/16:0. Thus, with ticagrelor or its active metabolite, the dynamics of the lipid composition of the platelet plasma membrane probably leads to an increase in membrane bending rigidity. Moreover, it has been shown that aspirin influences the conformation of membrane proteins, perhaps through the reduction in membrane lipid fluidity, rigidifying the lipid environment around the embedded proteins. Consequently, changes in the dynamics of the lipid bilayer could render some membrane receptors more exposed at the surface of the plasma membrane [42]. The lipid composition of the plasma membrane influences the deformability and the intrinsic curvature of this membrane and impacts the recruitment of specific proteins [29]. Other classes of drugs are responsible for an alteration of the whole lipid composition of the platelet. Lipid lowering drugs, such as statin drugs (i.e., fluvastatin) were shown to induce a decrease in the cholesterol/phospholipid ratio in platelets [47].

In our study, ticagrelor and its active metabolite modified the lipid composition in the plasma membrane. Concentrations of 1.5 µM ticagrelor and 0.5 µM active metabolite were chosen to be as close as possible to physiological blood concentrations found in treated patients. Indeed, patients with a dose regimen of 90 mg bid have a peak at 770 ng/mL for ticagrelor, and 257 ng/mL for its active metabolite, after 4 weeks [48]. Moreover, the ratio of three to one between ticagrelor and its active metabolite found in vivo has been also respected. Therefore, concentrations of 1.5 µM ticagrelor (i.e., 783.9 ng/mL), and 0.5 µM active metabolite (i.e., 239.3 ng/mL) were presumed to achieve effective platelet inhibition. Consequently, we used these concentrations for all subsequent lipidomics experiments. However, the use of a single concentration of ticagrelor and its metabolite remains a limitation of our study. Some specific lipid species with high biological functionality are impacted, especially ether lipids, which are subdivided into alkyl-ether and alkenyl-ether, the latter being also named plasmalogens [49]. Plasmalogens, especially plasmenylethanolamines, play a crucial role both for the structure of the cell membrane and cell signaling through the plasma membrane. These ether lipids form non-lamellar inverted hexagonal structures involved in facilitating fusion processes [50,51]. Moreover, ether lipids are important for the organization and the stability of cholesterol-enriched microdomains, and act as a platform in cellular signaling. Ether lipids and derivatives were also described as endogeneous antioxidants, directly implicated in cell differentiation and signaling pathways, such as AKT/PKB; PKC; PPAR; GPCR; and MAPK [52]. Plasmalogens including PUFA containing arachidonic acid in their acyl chains, especially PC O- 16:0/20:4, PC O- 18:1/20:4 or PE O- 16:1/20:4, PE O- 18:2/20:4 were significantly decreased for both molecules or for ticagrelor only, respectively. 

As reported by Wood et al., certain neuronal pathologies, such as schizophrenia, induce an alteration of the dynamics of plasmalogens in platelets [53]. Involvement of ether lipids in neuronal diseases is now well established in the literature [52,54,55] but the physiopathology remains incompletely elucidated. Our study highlights that drugs such as antiplatelet agents induced modifications in the lipid composition of the membrane, in particular in these ether lipids enriched in arachidonic acid, providing a source of arachidonic acid for phospholipase A2 hydrolysis [54,56]. Indeed, arachidonic acid is a substrate for the synthesis of biologically active mediators such as eicosanoids with prostaglandines, thromboxanes and leukotrienes, implicated in platelet aggregation and proinflammatory processes [57,58,59]. We can hypothesize that these changes may lead to a decrease in the potential mobilization of arachidonic acid from the membrane phospholipids for synthesis of biologically active mediators. This hypothesis warrants confirmation in further investigations especially through assessment of thromboxane B2 (TXB2) levels, the precursor of arachidonic acid [60]. However, the assessment of TXB2 levels was not initially included in our experimental protocol, which is a limitation of our study to confirm this hypothesis.

High arachidonic acid proportions promote inflammation, endothelial activation, leukocyte adhesion and thrombosis. Mobilization of arachidonic from diacyl phospholipid species (PC or PI) to ether-phospholipids, especially the ethanolamines plasmalogens, is due to the action of coenzyme A-independent transacylase [61,62]. A recent study showed that the coenzyme A-independent transacylase is not influenced by the membrane plasmalogen content to regulate the availability of arachidonic acid [61]. Nevertheless, arachidonylated plasmalogens were selectively hydrolyzed by cytosolic PLA2 in platelets [63,64]. Thus, these plasmalogens enriched in arachidonic acid play a crucial role in the platelets and could be mobilized by the cytosolic PLA2. The decrease in arachidonic acid in phosphatidyl-ether with ticagrelor and its active metabolite suggest that these drugs may be of clinical benefit in addition to their action on the P2Y12 receptor to reduce the availability of arachidonic acid by decreasing its proportion in plasmalogens.

## 4. Material and Methods

### 4.1. Experimental Groups

Human healthy platelets were studied in 6 experimental groups: (i) a control group, (ii) platelets activated by ADP 20 µM, (iii) platelets treated by ticagrelor 1.5 µM, (iv) platelets treated by ticagrelor active metabolite (AR-C124910XX, 0.5 µM), (v) platelets treated by ticagrelor 1.5 µM for 10 min then by ADP 20 µM for 10 min, and (vi) platelets treated by ticagrelor active metabolite 0.5 µM for 10 min then by ADP 20 µM for 10 min. After incubation of treatment for 10 min in a water bath at 37 °C, platelet plasma membranes were isolated using ultracentrifugation in a density gradient and subjected to mass spectrometry-based lipid analysis (Lipotype GmbH, Dresden, Germany).

### 4.2. Extraction of Platelet Plasma

Platelets were obtained from healthy donors from the French Blood Transfusion Center (Etablissement Français du Sang (EFS) Bourgogne Franche-Comté, Besançon, France). According to the agreement between the EFS and the Bourgogne Franche-Comté University (convention ref. DECO-15-0178), the EFS delivered anonymized samples after healthy adult blood donors gave written informed consent specifying the exclusive research purpose and the respect of ethical guidelines. Platelets were received in the laboratory for experiments on the same day as collection. For each replicate (n = 3), the 6 experimental conditions were performed on the same platelet concentrate from a pool of 5–7 donor units. Platelet pools were resuspended in solution of buffer A (90 mg Bovine Serum Albumin (BSA), 45 mg glucose, 50 mL 4-(2-hydroxyethyl)-1-piperazineethanesulfonicacid (HEPES), pH = 6.8) with prostagladin I2 to avoid platelet activation, followed by centrifugation at 2500 rpm. Supernatant was discarded, and a new wash with buffer A was performed. Then a third wash was performed with a solution of buffer B (45 mg glucose, 50 µL Ca^2+^ 1M, 50 mL HEPES) with apyrase into a water bath at 37 °C for 45 min. According to experimental groups, whole platelets were incubated with PBS (control group), ADP (ADP group, Bio/Data corporation, Horsham, PA, USA), ticagrelor (Ticagrelor group, Alsachim, Illkirch, France), ticagrelor active metabolite (Metabolite group, Alsachim, Illkirch, France), ticagrelor and ADP (Tica + ADP) and ticagrelor active metabolite and ADP (Meta + ADP) for 10 min in a water bath at 37 °C with regular gentle homogenization. After these incubations, a final centrifugation was performed for each condition to remove the supernatant. Each pellet was resuspended in PBS with antiprotease and EDTA, associated with gentle agitation. Dounce homogenization was used on whole platelet thus obtained to induce platelet lysis and were ultracentrifugated in sucrose gradient at 100,000 g for 2 h at 4 °C to remove cytosolic fractions and intracellular organelle membranes.

### 4.3. VASP Phosphorylation Analysis

VASP phosphorylation analysis was performed using VASP kits (BioCytex, Marseille, France) within 48 h after blood sampling collected on sodium citrate. Blood samples were incubated in vitro with ADP and/or prostaglandin E1 (PGE1) before fixation, according to the manufacturer’s instructions. Indirect immunolabeling on each sample was performed with a first incubation with 16C2 monoclonal antibody, followed by staining with a goat antimouse fluorescein isothiocyanate polyclonal reagent (BioCytex, Marseille, France). Flow cytometric analysis was performed on a NAVIOS cytometer (Beckman Coulter Life Sciences Inc., Brea, CA, USA). The platelet population was identified by its forward and side scatter distribution, and 3000 platelet events were gated and analyzed for mean fluorescence intensity (MFI), using NAVIOS software. An MFI corresponding to each experimental condition (ADP, ADP + PGE1) was determined to establish a ratio that was directly correlated with VASP phosphorylation state. A Platelet Reactivity Index (PRI VASP) was calculated from the MFI of samples incubated with PGE1 or PGE1 and ADP according to the formula: PRI VASP = [(MFI(PGE1) − MFI(PGE1) + ADP)/MFI(PGE1)] × 100. The PRI is expressed as a percentage. The normal value of the PRI in healthy subjects is higher than 70% [12]. Efficient inhibition of platelets is obtained with a PRI below 50% [65].

### 4.4. Immunoblot Assay

In order to determine the position of platelet plasma membrane in sucrose fraction, the presence of specific protein markers was evaluated by immunoblot assay in the control group.

Rabbit monoclonal anti-Flotillin-1 (ab411927, Abcam, Paris, France) anti-GpIb (ab192541, Abcam, Paris, France), anti-NCF1 (ab181090, Abcam, Paris, France) and rabbit polyclonal anti-COX IV (ab16056, Abcam, Paris, France) were used as markers of cholesterol rich microdomains, platelet plasma membrane, cytosol and mitochondria, respectively. 

Protein levels in each fraction were quantified by BCA Protein Assay (Thermo Scientific France, Villebon sur Yvette, France). Three microliters of each fraction, containing the same quantity of proteins, were put on nitrocellulose membrane directly. After blocking non-specific binding sites for 1 h with BSA 5% in Tris Buffered Saline (TBS)-Tween (TBS with 0.1% Tween 20), membranes were incubated by primary antibodies, and horseradish peroxidase conjugated goat anti rabbit IgG Heavy & Light (ab6721, Abcam, Paris, France) was used as secondary antibodies.

### 4.5. Lipid Extraction for Mass Spectrometry Lipidomics

Mass spectrometry-based lipid analysis was performed by Lipotype GmbH (Dresden, Germany) as previously described [66]. Lipids were extracted from samples (n = 3 for each experimental group) using a two-step chloroform/methanol procedure [67]. Samples were spiked with internal lipid standard mixture containing: cardiolipin 16:1/15:0/15:0/15:0 (CL), ceramide 18:1;2/17:0 (Cer), diacylglycerol 17:0/17:0 (DAG), hexosylceramide 18:1;2/12:0 (HexCer), lyso-phosphatidate 17:0 (LPA), lyso-phosphatidylcholine 12:0 (LPC), lyso-phosphatidylethanolamine 17:1 (LPE), lyso-phosphatidylglycerol 17:1 (LPG), lyso-phosphatidylinositol 17:1 (LPI), lyso-phosphatidylserine 17:1 (LPS), phosphatidate 17:0/17:0 (PA), phosphatidylcholine 17:0/17:0 (PC), phosphatidylethanolamine 17:0/17:0 (PE), phosphatidylglycerol 17:0/17:0 (PG), phosphatidylinositol 16:0/16:0 (PI), phosphatidylserine 17:0/17:0 (PS), cholesterol ester 20:0 (CE), sphingomyelin 18:1;2/12:0;0 (SM), triacylglycerol 17:0/17:0/17:0 (TAG) and cholesterol D6 (Chol). After extraction, the organic phase was transferred to an infusion plate and dried in a speed vacuum concentrator. The first step dry extract was re-suspended in 7.5 mM ammonium acetate in chloroform/methanol/propanol (1:2:4, V:V:V) and the 2nd step dry extract in a 33% ethanol solution of methylamine in chloroform/methanol (0.003:5:1; V:V:V). All liquid handling steps were performed using the Hamilton Robotics STARlet robotic platform with the Anti Droplet Control feature for organic solvent pipetting. 

### 4.6. MS Data Acquisition

Lipid extracts (n = 3 for each experimental group) were analyzed by direct infusion on a QExactive mass spectrometer (Thermo Scientific, Osterode an Harz, Germany) equipped with a TriVersa NanoMate ion source (Advion Biosciences, Ithaca, NY, USA). Samples were analyzed in both positive and negative ion modes with a resolution of Rm/z = 200 = 280,000 for MS and Rm/z = 200 = 17,500 for MSMS experiments, in a single acquisition. MSMS was triggered by an inclusion list encompassing corresponding MS mass ranges scanned in 1 Da increments [68]. Both MS and MSMS data were combined to monitor CE, DAG and TAG ions as ammonium adducts; PC, PC O-, as acetate adducts; and CL, PA, PE, PE O-, PG, PI and PS as deprotonated anions. MS only was used to monitor LPA, LPE, LPE O-, LPI and LPS as deprotonated anions; Cer, HexCer, SM, LPC and LPC O- as acetate adducts and cholesterol as the ammonium adduct of an acetylated derivative [69].

### 4.7. Data Analysis and Post-Processing

Data were analyzed with a specifically developed lipid identification software based on LipidXplorer [70,71]. Data post-processing and normalization were performed using a developed data management system. Limits of quantification are in the lower µM to sub-µM range, depending on the lipid class. Only lipid identifications with a signal-to-noise ratio >5, and a signal intensity 5-fold higher than in corresponding blank samples were considered for further data analysis. 

### 4.8. Statistical Analysis

Data are expressed as mean ± SD. Significant differences among experimental groups were determined by one-way or two ANOVA tests. A *p*-value < 0.05 was considered significant. All tests were performed using GraphPad (GraphPad Software Inc., San Diego, CA, USA) and SciPy (SciPy.org).

## 5. Conclusions

In conclusion, this study details for the first time the lipid composition of the plasma membrane of resting platelets using advances in lipidomics. Moreover, this study shows that antiplatelet agents, such as ticagrelor and its active metabolite, modify the lipid composition of platelet plasma membrane. Our results suggest that ticagrelor and its active metabolite have a long-lasting impact on the lipid composition of the platelet plasma membrane. Thus, ticagrelor and its active metabolite could impact the lipid environment of the receptor embedded in the lipid bilayer and modify the behavior of the plasma membrane. Further studies will be required to investigate the relationship between ticagrelor and the functional impact of alternations in the lipid composition for the platelet plasma membrane in terms of regulation of platelet aggregability and therapeutic implications. In this regard, molecular dynamics simulations appear to be a useful tool to shed light on this new aspect of lipid–protein interactions. This work is currently in progress.

## Figures and Tables

**Table 1:** Results of our lipid composition of plasma membrane of resting platelet or whole resting platelets compared to the previous lipid profile of the platelet described.

	Cholesterol (%)	PC and Related Species (%)		PE and Related Species (%)		PI (%)	PS (%)	SM (%)	Other Lipids Species	Method	Ref/Years
		PC	PC O-	PE	PE O-						
**Plasma membrane of resting platelet**	Unknown	35	-	26.1	-	5.6	9.9	23.4		Two-dimensional thin layer chromatography and gas–liquid chromatography	Perret et al. [13]/1979
	Unknown	39.2	-	27.8	-	2.5	8.6	22.1		Thin layer chromatography and gas–liquid chromatography after transmethylation	Skeaff et al. [14]/1985
	7.5	34.8	-	25.3	-	5.8	6.4	14.6	5.2	Rod like thin-layer chromatography	Tsvetkova et al. [15]/1999
	Unknown	27	-	29	-	2	19	22	-	High performance thin layer chromatography	Biró et al. [16]/2005
	**28.35**	**37.01**		**16.81**		**2.41**	**6.06**	**7.28**	**2.08**	**Mass spectrometry**	**Result of our study**
		**35.17**	**1.84**	**6.41**	**10.40**						
	**Without cholesterol**	**51.92**		**23.59**		**3.38**	**8.50**	**10.19**	**2.42**	**Mass spectrometry**	**Result of our study**
		49.34	2.58	9.00	14.59						
**Lipid composition of whole resting platelet**	Unknown	39.8	1.7	13.6	14.0	3.7	8.8	18.4		Thin layer and gas–liquid chromatography	Cohen et al. [17]/1969
	Unknown	38.56	-	29.12	-	4.17	10.98	14.96	2.21	Two-dimensional thin layer chromatography and gas chromatography	Broekman et al. [18]/1980
	Unknown	38.7	-	26.9	-	4.4	9.8	18.5	1.7	Thin-layer chromatography and gas–liquid chromatography	Hamid et al. [19]/1980
	31.5	27.5	-	18.9	-	1.8	6.3	13.1	0.9	Two-dimensional thin layer chromatography	Owen et al. [20]/1981
	18.9	34.8	-	18.8	-	11.8	13.2	16.0		Thin layer and gas–liquid chromatography	In Italy, Dougherty et al. [21]/1987
	22.2	38.2	-	25.8	-	6.5	10.6	13.2		Thin layer and gas–liquid chromatography	In Finland, Dougherty et al. [21]/1987
	19.3	31.1	-	23	-	10.6	12.0	16.5		Thin layer and gas–liquid chromatography	In USA, Dougherty et al. [21]/1987
	15,15	43	-	21.47	-	8.8		26	-	thin-layer chromatography using a plate scanner with a flame ionization detector	Watanabe et al. [22]/1998
	30	26	-	12	-	1	9	11	2	Mass spectrometry	Leidl et al. [23]/2008
**Lipid composition of whole stimulated platelet by thrombin**	Unknown	38.44	-	28.96	-	3.00	11.01	15.1	3.49	Two-dimensional thin layer chromatography and gas chromatography	Broekman et al. [18]/1980
	Unknown	36.2	-	29.4	-	1.6	9.3	23.6	-	Gas–liquid chromatography	Skeaff et al. [14]/1985

Our results are presented in bold, with phosphatidylcholines (PC); phosphatidylcholines ether (PC O-); phosphatidylethanolamines (PE); phosphatidylethanolamines ether (PE O-); phosphatidylinositols (PI); phosphatidylserines (PS) and sphingomyelins (SM). Complete values are available in Supplementary Materials Table S1.

## Data Availability

The data presented in this study are openly available in Supplementary materials Table S1. Further inquiries can be directed to the corresponding author.

## Supplementary Materials

Supplementary materials can be found at https://www.mdpi.com/1422-0067/22/3/1432/s1.

## Abbreviations

ADP	Adenosine diphosphate
BSA	Bovine Serum Albumin
CL	Cardiolipin
Cer	Ceramide
Chol	Cholesterol
CE	Cholesteryl ester
COX IV	Cytochrome c oxidase IV
DAG	Diacylglycerol
DHA	Docosahexaenoic acid
EPA	Eicosapentaenoic acid
ENT1	Equilibrative Nucleoside Transporter 1
GP1b	Glycoprotein Ib
HexCer	Hexosylceramide
LPA	Lyso-phosphatidates
LPC	Lyso-phosphatidylcholines
LPE	Lyso-phosphatidylethanolamines
LPG	Lyso-phosphatidylglycerols
LPI	Lyso-phosphatidylinositols
LPS	Lyso-phosphatidylserines
MFI	Mean Fluorescence Intensity
NCF1	Neutrophil cytosolic factor 1
PA	Phosphatidate
PCA	Principal Component Analysis
PC	Phosphatidylcholines
PC O-	Phosphatidylcholines ether
PE	Phosphatidylethanolamines
PE O-	Phosphatidylethanolamines ether
PG	Phosphatidylglycerols
PI	Phosphatidylinositols
PS	Phosphatidylserines
PGE1	Prostaglandin E1
PRI	Platelet Reactivity Index
PUFA	Polyunsaturated Fatty Acids
SM	Sphingomyelins
TAG	Triacylglycerol
VASP	Vasodilator-Stimulated Phosphoprotein

## References

[1] Jurk K., Kehrel B.E. (2005). Platelets: Physiology and Biochemistry. Semin. Thromb. Hemost..

[2] Thomas S.G., Michelson A.D. (2019). 3-The Structure of Resting and Activated Platelets. Platelets.

[3] von Kügelgen I. (2017). Structure, Pharmacology and Roles in Physiology of the P2Y12 Receptor. Adv. Exp. Med. Biol..

[4] Valgimigli M., Bueno H., Byrne R.A., Collet J.-P., Costa F., Jeppsson A., Jüni P., Kastrati A., Kolh P., Mauri L. (2018). 2017 ESC Focused Update on Dual Antiplatelet Therapy in Coronary Artery Disease Developed in Collaboration with EACTS: The Task Force for Dual Antiplatelet Therapy in Coronary Artery Disease of the European Society of Cardiology (ESC) and of the European Association for Cardio-Thoracic Surgery (EACTS). Eur. Heart J..

[5] Cammisotto V., Biondi-Zoccai G., Frati G., Giordano A. (2019). Prasugrel and Ticagrelor: The Romulus and Remus of Antiplatelet Therapy?. Am. J. Cardiovasc Drugs.

[6] Nylander S., Schulz R. (2016). Effects of P2Y12 Receptor Antagonists beyond Platelet Inhibition—Comparison of Ticagrelor with Thienopyridines. Br. J. Pharm..

[7] Aungraheeta R., Conibear A., Butler M., Kelly E., Nylander S., Mumford A., Mundell S.J. (2016). Inverse Agonism at the P2Y12 Receptor and ENT1 Transporter Blockade Contribute to Platelet Inhibition by Ticagrelor. Blood.

[8] Rabani V., Montange D., Meneveau N., Davani S. (2017). Impact of Ticagrelor on P2Y1 and P2Y12 Localization and on Cholesterol Levels in Platelet Plasma Membrane. Platelets.

[9] Rawicz W., Olbrich K.C., McIntosh T., Needham D., Evans E. (2000). Effect of Chain Length and Unsaturation on Elasticity of Lipid Bilayers. Biophys. J..

[10] Peng B., Geue S., Coman C., Münzer P., Kopczynski D., Has C., Hoffmann N., Manke M.-C., Lang F., Sickmann A. (2018). Identification of Key Lipids Critical for Platelet Activation by Comprehensive Analysis of the Platelet Lipidome. Blood.

[11] O’Donnell V.B., Murphy R.C., Watson S.P. (2014). Platelet Lipidomics: Modern Day Perspective on Lipid Discovery and Characterization in Platelets. Circ. Res..

[12] Schwarz U.R., Geiger J., Walter U., Eigenthaler M. (1999). Flow Cytometry Analysis of Intracellular VASP Phosphorylation for the Assessment of Activating and Inhibitory Signal Transduction Pathways in Human Platelets—Definition and Detection of Ticlopidine/Clopidogrel Effects. Thromb. Haemost..

[13] Perret B., Chap H.J., Douste-Blazy L. (1979). Asymmetric Distribution of Arachidonic Acid in the Plasma Membrane of Human Platelets. A Determination Using Purified Phospholipases and a Rapid Method for Membrane Isolation. Biochim. Biophys. Acta.

[14] Skeaff C.M., Holub B.J. (1985). Altered Phospholipid Composition of Plasma Membranes from Thrombin-Stimulated Human Platelets. Biochim. Biophys. Acta.

[15] Tsvetkova N.M., Crowe J.H., Walker N.J., Crowe L.M., Oliver A.E., Wolkers W.F., Tablin F. (1999). Physical Properties of Membrane Fractions Isolated from Human Platelets: Implications for Chilling Induced Platelet Activation. Mol. Membr. Biol..

[16] Biró E., Akkerman J.W.N., Hoek F.J., Gorter G., Pronk L.M., Sturk A., Nieuwland R. (2005). The Phospholipid Composition and Cholesterol Content of Platelet-Derived Microparticles: A Comparison with Platelet Membrane Fractions. J. Thromb. Haemost..

[17] Cohen P., Derksen A. (1969). Comparison of Phospholipid and Fatty Acid Composition of Human Erythrocytes and Platelets. Br. J. Haematol..

[18] Broekman M.J., Ward J.W., Marcus A.J. (1980). Phospholipid Metabolism in Stimulated Human Platelets. Changes in Phosphatidylinositol, Phosphatidic Acid, and Lysophospholipids. J. Clin. Investig..

[19] Hamid M.A., Kunicki T.J., Aster R.H. (1980). Lipid Composition of Freshly Prepared and Stored Platelet Concentrates. Blood.

[20] Owen J.S., Hutton R.A., Day R.C., Bruckdorfer K.R., McIntyre N. (1981). Platelet Lipid Composition and Platelet Aggregation in Human Liver Disease. J. Lipid Res..

[21] Dougherty R.M., Galli C., Ferro-Luzzi A., Iacono J.M. (1987). Lipid and Phospholipid Fatty Acid Composition of Plasma, Red Blood Cells, and Platelets and How They Are Affected by Dietary Lipids: A Study of Normal Subjects from Italy, Finland, and the USA. Am. J. Clin. Nutr.

[22] Watanabe M., Shiraishi K., Itakura M., Matsuzaki S. (1998). Relationship between Platelet Membrane Lipid Compositions and Platelet Aggregability in Alcoholic Liver Disease. Alcohol Clin. Exp. Res..

[23] Leidl K., Liebisch G., Richter D., Schmitz G. (2008). Mass Spectrometric Analysis of Lipid Species of Human Circulating Blood Cells. Biochim. Et Biophys. Acta (BBA) Mol. Cell Biol. Lipids.

[24] Ruebsaamen K., Liebisch G., Boettcher A., Schmitz G. (2010). Lipidomic Analysis of Platelet Senescence. Transfus..

[25] Yawata Y. (2003). Cell Membrane: The Red Blood Cell as a Model..

[26] Lorent J.H., Levental K.R., Ganesan L., Rivera-Longsworth G., Sezgin E., Doktorova M., Lyman E., Levental I. (2020). Plasma Membranes Are Asymmetric in Lipid Unsaturation, Packing and Protein Shape. Nat. Chem. Biol..

[27] Subczynski W.K., Pasenkiewicz-Gierula M., Widomska J., Mainali L., Raguz M. (2017). High Cholesterol/Low Cholesterol: Effects in Biological Membranes Review. Cell Biochem. Biophys..

[28] Baum S.J., Kris-Etherton P.M., Willett W.C., Lichtenstein A.H., Rudel L.L., Maki K.C., Whelan J., Ramsden C.E., Block R.C. (2012). Fatty Acids in Cardiovascular Health and Disease: A Comprehensive Update. J. Clin. Lipidol.

[29] Harayama T., Riezman H. (2018). Understanding the Diversity of Membrane Lipid Composition. Nat. Rev. Mol. Cell Biol..

[30] Pinot M., Vanni S., Pagnotta S., Lacas-Gervais S., Payet L.-A., Ferreira T., Gautier R., Goud B., Antonny B., Barelli H. (2014). Lipid Cell Biology. Polyunsaturated Phospholipids Facilitate Membrane Deformation and Fission by Endocytic Proteins. Science.

[31] McMahon H.T., Boucrot E. (2015). Membrane Curvature at a Glance. J. Cell Sci..

[32] Sezgin E., Levental I., Mayor S., Eggeling C. (2017). The Mystery of Membrane Organization: Composition, Regulation and Roles of Lipid Rafts. Nat. Rev. Mol. Cell Biol..

[33] Rabani V., Lagoutte-Renosi J., Series J., Valot B., Xuereb J.-M., Davani S. (2020). Cholesterol-Rich Microdomains Contribute to PAR1 Signaling in Platelets Despite a Weak Localization of the Receptor in These Microdomains. Int. J. Mol. Sci..

[34] Rabani V., Montange D., Davani S. (2016). Interactive Protein Network of FXIII-A1 in Lipid Rafts of Activated and Non-Activated Platelets. Platelets.

[35] Corradi V., Sejdiu B.I., Mesa-Galloso H., Abdizadeh H., Noskov S.Y., Marrink S.J., Tieleman D.P. (2019). Emerging Diversity in Lipid-Protein Interactions. Chem. Rev..

[36] Prescott S.M., Majerus P.W. (1981). The Fatty Acid Composition of Phosphatidylinositol from Thrombin-Stimulated Human Platelets. J. Biol. Chem..

[37] Prisco D., Tufano A., Cenci C., Pignatelli P., Santilli F., Di Minno G., Perticone F. (2019). Position Paper of the Italian Society of Internal Medicine (SIMI) on Prophylaxis and Treatment of Venous Thromboembolism in Patients with Cancer. Intern. Emerg Med..

[38] Ren H., Okpala I., Ghebremeskel K., Ugochukwu C.C., Ibegbulam O., Crawford M. (2005). Blood Mononuclear Cells and Platelets Have Abnormal Fatty Acid Composition in Homozygous Sickle Cell Disease. Ann. Hematol.

[39] García-Rubio D., Rodríguez-Varela M., Martínez-Vieyra I., de la Mora M.B., Méndez-Méndez J.V., Durán-Álvarez J.C., Cerecedo D. (2019). Alterations to the Contents of Plasma Membrane Structural Lipids Are Associated with Structural Changes and Compartmentalization in Platelets in Hypertension. Exp. Cell Res..

[40] Sengupta D., Chattopadhyay A. (2015). Molecular Dynamics Simulations of GPCR-Cholesterol Interaction: An Emerging Paradigm. Biochim Biophys Acta.

[41] Mahmood I., Liu X., Neya S., Hoshino T. (2013). Influence of Lipid Composition on the Structural Stability of G-Protein Coupled Receptor. Chem. Pharm Bull. (Tokyo).

[42] Watała C., Gwoździński K. (1993). Effect of Aspirin on Conformation and Dynamics of Membrane Proteins in Platelets and Erythrocytes. Biochem. Pharm..

[43] Alves A.C., Ribeiro D., Horta M., Lima J.L.F.C., Nunes C., Reis S. (2017). A Biophysical Approach to Daunorubicin Interaction with Model Membranes: Relevance for the Drug’s Biological Activity. J. R Soc. Interface.

[44] Pham V.T., Nguyen T.Q., Dao U.P.N., Nguyen T.T. (2018). On the Interaction between Fluoxetine and Lipid Membranes: Effect of the Lipid Composition. Spectrochim. Acta A Mol. Biomol. Spectrosc..

[45] Winocour P.D., Watala C., Perry D.W., Kinlough-Rathbone R.L. (1992). Decreased Platelet Membrane Fluidity Due to Glycation or Acetylation of Membrane Proteins. Thromb. Haemost..

[46] Chakraborty S., Doktorova M., Molugu T.R., Heberle F.A., Scott H.L., Dzikovski B., Nagao M., Stingaciu L.-R., Standaert R.F., Barrera F.N. (2020). How Cholesterol Stiffens Unsaturated Lipid Membranes. Proc. Natl. Acad. Sci. USA.

[47] Osamah H., Mira R., Sorina S., Shlomo K., Michael A. (1997). Reduced Platelet Aggregation after Fluvastatin Therapy Is Associated with Altered Platelet Lipid Composition and Drug Binding to the Platelets. Br. J. Clin. Pharm..

[48] Storey R.F., Husted S., Harrington R.A., Heptinstall S., Wilcox R.G., Peters G., Wickens M., Emanuelsson H., Gurbel P., Grande P. (2007). Inhibition of Platelet Aggregation by AZD6140, a Reversible Oral P2Y12 Receptor Antagonist, Compared with Clopidogrel in Patients with Acute Coronary Syndromes. J. Am. Coll Cardiol..

[49] Jiménez-Rojo N., Riezman H. (2019). On the Road to Unraveling the Molecular Functions of Ether Lipids. FEBS Lett..

[50] Dean J.M., Lodhi I.J. (2018). Structural and Functional Roles of Ether Lipids. Protein Cell.

[51] Koivuniemi A. (2017). The Biophysical Properties of Plasmalogens Originating from Their Unique Molecular Architecture. FEBS Lett..

[52] Dorninger F., Forss-Petter S., Wimmer I., Berger J. (2020). Plasmalogens, Platelet-Activating Factor and beyond—Ether Lipids in Signaling and Neurodegeneration. Neurobiol Dis.

[53] Wood P.L., Unfried G., Whitehead W., Phillipps A., Wood J.A. (2015). Dysfunctional Plasmalogen Dynamics in the Plasma and Platelets of Patients with Schizophrenia. Schizophr. Res..

[54] Braverman N.E., Moser A.B. (2012). Functions of Plasmalogen Lipids in Health and Disease. Biochim. Et Biophys. Acta (BBA) Mol. Basis Dis..

[55] Meletis C.D. (2020). Alkyl-Acylglycerols and the Important Clinical Ramifications of Raising Plasmalogens in Dementia and Alzheimer’s Disease. Integr. Med. (Encinitas).

[56] Astudillo A.M., Balboa M.A., Balsinde J. (2019). Selectivity of Phospholipid Hydrolysis by Phospholipase A2 Enzymes in Activated Cells Leading to Polyunsaturated Fatty Acid Mobilization. Biochim. Biophys. Acta Mol. Cell Biol. Lipids.

[57] Brash A.R. (2001). Arachidonic Acid as a Bioactive Molecule. J. Clin. Investig..

[58] Sonnweber T., Pizzini A., Nairz M., Weiss G., Tancevski I. (2018). Arachidonic Acid Metabolites in Cardiovascular and Metabolic Diseases. Int. J. Mol. Sci..

[59] Tallima H., El Ridi R. (2017). Arachidonic Acid: Physiological Roles and Potential Health Benefits—A Review. J. Adv. Res..

[60] Hamberg M., Svensson J., Samuelsson B. (1975). Thromboxanes: A New Group of Biologically Active Compounds Derived from Prostaglandin Endoperoxides. Proc. Natl. Acad. Sci. USA.

[61] Lebrero P., Astudillo A.M., Rubio J.M., Fernández-Caballero L., Kokotos G., Balboa M.A., Balsinde J. (2019). Cellular Plasmalogen Content Does Not Influence Arachidonic Acid Levels or Distribution in Macrophages: A Role for Cytosolic Phospholipase A2γ in Phospholipid Remodeling. Cells.

[62] Yamashita A., Hayashi Y., Nemoto-Sasaki Y., Ito M., Oka S., Tanikawa T., Waku K., Sugiura T. (2014). Acyltransferases and Transacylases That Determine the Fatty Acid Composition of Glycerolipids and the Metabolism of Bioactive Lipid Mediators in Mammalian Cells and Model Organisms. Prog. Lipid Res..

[63] Nieto M.L., Venable M.E., Bauldry S.A., Greene D.G., Kennedy M., Bass D.A., Wykle R.L. (1991). Evidence That Hydrolysis of Ethanolamine Plasmalogens Triggers Synthesis of Platelet-Activating Factor via a Transacylation Reaction. J. Biol. Chem..

[64] Beckett C.S., Kell P.J., Creer M.H., McHowat J. (2007). Phospholipase A2-Catalyzed Hydrolysis of Plasmalogen Phospholipids in Thrombin-Stimulated Human Platelets. Thromb. Res..

[65] Freynhofer M.K., Brozovic I., Bruno V., Farhan S., Vogel B., Jakl G., Willheim M., Hübl W., Wojta J., Huber K. (2011). Multiple Electrode Aggregometry and Vasodilator Stimulated Phosphoprotein-Phosphorylation Assay in Clinical Routine for Prediction of Postprocedural Major Adverse Cardiovascular Events. Thromb. Haemost..

[66] Sampaio J.L., Gerl M.J., Klose C., Ejsing C.S., Beug H., Simons K., Shevchenko A. (2011). Membrane Lipidome of an Epithelial Cell Line. Proc. Natl. Acad. Sci. USA.

[67] Ejsing C.S., Sampaio J.L., Surendranath V., Duchoslav E., Ekroos K., Klemm R.W., Simons K., Shevchenko A. (2009). Global Analysis of the Yeast Lipidome by Quantitative Shotgun Mass Spectrometry. Proc. Natl. Acad. Sci. USA.

[68] Surma M.A., Herzog R., Vasilj A., Klose C., Christinat N., Morin-Rivron D., Simons K., Masoodi M., Sampaio J.L. (2015). An Automated Shotgun Lipidomics Platform for High Throughput, Comprehensive, and Quantitative Analysis of Blood Plasma Intact Lipids. Eur. J. Lipid Sci. Technol..

[69] Liebisch G., Binder M., Schifferer R., Langmann T., Schulz B., Schmitz G. (2006). High Throughput Quantification of Cholesterol and Cholesteryl Ester by Electrospray Ionization Tandem Mass Spectrometry (ESI-MS/MS). Biochim. Biophys. Acta.

[70] Herzog R., Schwudke D., Schuhmann K., Sampaio J.L., Bornstein S.R., Schroeder M., Shevchenko A. (2011). A Novel Informatics Concept for High-Throughput Shotgun Lipidomics Based on the Molecular Fragmentation Query Language. Genome Biol..

[71] Herzog R., Schuhmann K., Schwudke D., Sampaio J.L., Bornstein S.R., Schroeder M., Shevchenko A. (2012). LipidXplorer: A Software for Consensual Cross-Platform Lipidomics. PLoS ONE.

